# Perspectives of European Nutrition Professionals on Healthy Eating Policies: An International Comparative Study of Implementation Gaps and Information Credibility

**DOI:** 10.3390/nu18213493

**Published:** 2026-10-24

**Authors:** Paweł Bryła, Beata Ciabiada-Bryła

**Affiliations:** 1 Department of International Marketing and Retailing, University of Lodz, 90-131 Łódź, Poland; 2 Department of Preventive Medicine, Medical University of Lodz, 92-213 Lodz, Poland; beata.ciabiada-bryla@umed.lodz.pl

**Keywords:** healthy eating policies, dietitians, nutrition education, policy implementation, cross-national comparison, information credibility

## Abstract

**Background/Objectives:** This study compared the opinions of nutrition professionals from Poland, France, the United Kingdom, and Germany regarding the perceived importance and perceived use of policy instruments, audience characteristics affecting policy effectiveness, and credibility of nutrition information sources. **Methods:** A cross-sectional survey was completed by 131 nutrition professionals (56 Poland, 36 France, 26 UK, 13 Germany), who rated 14 policy instruments, 11 audience characteristics, and 9 information sources. Data were analyzed using ANOVA, ANCOVA, post-hoc tests with Bonferroni correction, and analysis of open-ended responses. **Results:** Across policy instruments, perceived importance consistently exceeded perceived use, most strongly for nutrition education for children/adolescents (4.59 vs. 2.76, *p* < 0.001), information on product processing (3.92 vs. 2.44), dietitian recommendations (3.86 vs. 2.94), higher taxes on less healthy foods (3.46 vs. 2.50), and social marketing (3.60 vs. 3.19). After Benjamini–Hochberg correction, in ANCOVAs adjusted for age and professional experience, cross-national differences were observed in the assessment of credibility for health claims, nutrition claims, social marketing campaigns, cooking blogs, and nutrition apps. Qualitative findings emphasized the need for compulsory school-based nutrition education, better access to qualified professionals, and stricter regulation of food marketing and labelling. **Conclusions:** Nutrition professionals perceive a clear underutilization of key policy instruments, particularly educational and fiscal measures. Greater involvement of dietitians in context-sensitive policy design and implementation is recommended to bridge the gap between policy intentions and real-world effectiveness.

## 1. Introduction

The promotion of healthy eating has become a paramount public health priority across Europe, driven by the escalating burden of diet-related non-communicable diseases including obesity, type 2 diabetes, cardiovascular conditions, and certain cancers. The World Health Organization has consistently highlighted that unhealthy diets are among the leading risk factors for mortality and morbidity in the European region, contributing substantially to healthcare costs, productivity losses, and reduced quality of life [1]. In response, governments, international bodies, and civil society organizations have developed and implemented a wide array of policy instruments aimed at shaping food environments, influencing consumer choices, and ultimately improving dietary patterns at population level.

These policy instruments span a considerable spectrum, ranging from educational interventions and information provision to regulatory measures and fiscal policies. Nutrition education programs targeting children and adolescents, for instance, seek to build foundational knowledge and skills that can support lifelong healthy eating habits. Information-based tools such as health claims, nutrition claims, ingredient lists, and nutritional information panels aim to equip consumers with the transparency needed to make informed food choices. Social marketing is an important tool for promoting healthy eating [2]. Recently, digital instruments including nutrition apps for smartphones and cooking blogs have emerged as increasingly influential sources of dietary guidance. At the regulatory end of the spectrum, policies such as higher taxes on less healthy food products, restrictions on marketing to children, and mandatory labelling of product processing levels represent more interventionist approaches to shaping food environments. The NOURISHING framework developed by the World Cancer Research Fund International [3] provides a comprehensive taxonomy of such policy instruments, encompassing food environment, food system, and behavior change communication interventions.

Despite the proliferation of healthy eating policies across Europe, evidence regarding their effectiveness remains mixed, and questions persist about the factors that facilitate or impede successful implementation. Nguyen et al. [4] in a systematic review of food environment policies implemented at scale, identified that stakeholder engagement, policy prioritization, and adequate resourcing were among the most commonly cited enablers of successful implementation, while concerns over profitability and revenue, together with insufficient political will, represented significant barriers. These findings underscore that the mere existence of policies is insufficient; their design, implementation context, and alignment with target audience characteristics all critically shape their ultimate impact.

An underexplored dimension in this policy landscape is the perspective of frontline nutrition professionals who work directly with individuals and communities to translate dietary guidance into practice. Dietitians occupy a unique position at the intersection of clinical practice, public health, and food policy. They possess specialized expertise in nutrition science, understand the practical challenges that individuals face in adopting healthier eating patterns, and observe at close quarters the strengths and limitations of existing policy instruments. As such, dietitians represent an invaluable source of insight for evaluating current policies and identifying priorities for future development. Their professional judgments about which instruments are most important, how well existing policies are being implemented, and what characteristics of target audiences matter most for effectiveness can inform evidence-based policy making and help bridge the gap between policy intentions and realized outcomes. Dietitians are recognized as experts in nutrition care and play a vital role in multidisciplinary healthcare teams [5]. Their specialized knowledge ensures that dietary guidelines and policies are based on the latest scientific evidence and best practices in nutrition. Dietitians advocate for a total diet approach, emphasizing balance, moderation, and variety rather than labelling specific foods as good or bad [6]. This perspective helps in creating more comprehensive and realistic dietary guidelines that can be more easily adopted by the general population. Dietitians’ insights are essential in addressing public health concerns such as obesity, malnutrition, and chronic diseases. Their involvement in policy-making can lead to more effective interventions that promote healthy eating habits and improve overall public health outcomes [7,8]. Dietitians understand the cultural and social factors that influence eating behaviors. Their input can help ensure that healthy eating policies are culturally acceptable and consider the diverse needs of different population groups [9,10]. Dietitians are involved in the practical aspects of nutrition education and food service management. Their experience in these areas can provide valuable insights into the feasibility and effectiveness of proposed policies [11]. Dietitians use evidence-based strategies and behavioral theories to promote sensible food choices. Their understanding of the psychological and social determinants of eating behaviors can help in designing policies that are more likely to be accepted and followed by the public [12]. Dietitians often work closely with other healthcare providers, which can enhance the multidisciplinary approach to healthcare and ensure that nutrition is integrated into overall health management. In summary, the opinions of dietitians are vital in shaping healthy eating policies that are scientifically sound, culturally sensitive, practical, and sustainable. Their expertise and holistic approach can significantly contribute to the development of effective public health strategies that promote better nutrition and overall well-being. In another study stemming from the same research project, we compared European dietitians’ opinions on communication channels and priority topics for healthy eating campaigns targeting different age groups [13]. To date, no study has systematically compared the opinions of nutrition professionals across multiple European countries regarding the full spectrum of healthy eating policy instruments, their implementation, and the audience characteristics that influence their effectiveness.

This gap in the literature is significant for several reasons. First, the European Union comprises diverse national contexts with varying histories of nutrition policy, different healthcare and education systems, and distinct cultural orientations toward food and health. Understanding how nutrition professionals in different countries perceive policy instruments can reveal whether certain approaches command cross-national professional consensus or whether perspectives are shaped by local context. Such insights are essential for the development of European-level policy frameworks that respect national diversity while promoting coherent approaches to shared public health challenges. Second, the voice of dietitians is often absent from policy debates, yet their frontline experience positions them to identify implementation barriers and opportunities that may not be apparent to policymakers or researchers. Capturing and amplifying this professional perspective can enrich policy development and enhance the likelihood of successful implementation.

The present study addresses this gap by conducting an international comparative investigation of the opinions of nutrition professionals from four large European countries: Poland, France, the United Kingdom, and Germany. These countries were selected to represent different geographic regions within Europe, different healthcare system models, and different traditions of nutrition policy and public health intervention. The study pursued four specific objectives: first, to examine nutrition professionals’ assessments of the importance of a comprehensive range of healthy eating policy instruments; second, to evaluate their perceptions of how extensively these instruments are currently used in their respective countries; third, to identify the characteristics of target audiences that nutrition professionals consider most important for policy effectiveness; and fourth, to assess the credibility that nutrition professionals attach to various types of nutrition and health-related information.

In addressing these objectives, the study also explored cross-national differences in professional perspectives, recognizing that the national context in which nutrition professionals practice may shape their judgments about policies, audiences, and information sources. Given the significant variation across the four countries in terms of policy histories, healthcare systems, and cultural orientations toward food, we anticipated finding meaningful differences in opinions of nutrition professionals. At the same time, we expected certain areas of consensus to emerge, reflecting shared professional values and common evidence bases.

The findings of this study have the potential to inform policy development at both national and European levels. By identifying which instruments nutrition professionals consider most important and which they perceive as underutilized, the research can highlight priorities for policy attention and resource allocation. By revealing the audience characteristics that professionals believe matter most for effectiveness, the study can guide targeting and tailoring of interventions. And by illuminating nutrition professionals’ credibility assessments of different information sources, the research can contribute to debates about how to strengthen the information environment and combat nutrition misinformation.

This article is structured as follows. The next section describes the material and methods employed in the study, including sampling procedures, data collection instrument, and analytical approaches. The results section presents findings organized around the study’s four objectives, incorporating both quantitative analyses and qualitative insights from open-ended questions. The discussion section interprets these findings in light of existing literature, considers their implications for policy and practice, and acknowledges the study’s limitations. The article concludes with a summary of key messages and recommendations for future research and policy development.

## 2. Materials and Methods

Our aim was to obtain opinions of nutrition professionals from four large European counties: Poland, France, UK, and Germany. We allowed dietitians and nutritionists to participate in this study. In Poland, the survey was addressed to all businesses classified in the “dietetics” category in Polish Telephone Directories (https://www.pkt.pl/szukaj/dietetyka?selectedCategoryRefinement=Dietetyka (accessed on 31 July 2024)), as well as numerous Facebook groups of dietitians and some LinkedIn groups. Moreover, a post with the invitation to participate in the survey was put on the https://dietetycy.org.pl/ (accessed on 13 January 2025), portal, and an email request was sent to the Polish Society of Dietetics. In France, a database with contact details of nutrition professionals was purchased from Base-Emails.com. In the UK, contact questionnaires were used at https://www.bda.uk.com/find-a-dietitian.html (accessed on 1 September 2025), https://www.nutritionist-resource.org.uk/ (accessed on 1 September 2025), and https://practitioner-search.bant.org.uk/ (accessed on 1 September 2025). In Germany, the emails of nutrition professionals were found at https://www.vdd.de/diaetassistenten-suche/ (accessed on 31 July 2024). Overall, we have received 131 completed questionnaires, including 56 in Poland, 36 in France, 26 in the UK, and 13 in Germany. The selection of instruments was partly based on previous own research [14] and was supplemented with recent literature review about healthy eating policies. It was refined as a result of discussion among members of the research team (Opus grant no. 2023/49/B/HS5/00127). More details about the questionnaire design and survey execution are available in a previous article stemming from the same research project [13]. The dataset has been deposited in Zenodo [15].

Data analysis was conducted in IBM SPSS 29. For nominal variables, we applied cross-tabulation and Chi-square tests. To account for major differences in some characteristics of respondents across the national samples, means were compared with the use of one-way analysis of covariance (ANCOVA), adjusted for age and professional experience, and post-hoc tests with Bonferroni correction were applied to identify between which countries there were significant differences. To account for the multiplicity of statistical tests, we controlled the false discovery rate using the Benjamini–Hochberg (BH) procedure with a 5% significance level. This approach was chosen to balance the risk of Type I errors with the need to identify meaningful cross-national differences in this exploratory comparative study. To compare importance attached to healthy eating policy instruments with assessment of their use in one’s country, we applied *t*-tests for dependent samples.

As ANCOVA assumptions, we tested homogeneity of regression slopes (checking if the interactions between the independent variable and each covariate are not significant) and homogeneity of variance (with the use of the Levene’s test). ANCOVA’s assumptions (*p*s > 0.05) were met for all analyses concerning opinions about the importance of selective instruments for the effective promotion of healthy eating (Table A1), with the exception of the Levene’s test for nutrition education. Similarly to the Levene’s test, the Breusch-Pagan test for heteroscedasticity was also significant for this variable (***p* < 0.001**). As far as the opinions about the level of use of selected healthy eating policy instruments in one’s country are concerned, assumptions were met with the exceptions of a significant interaction term of country with experience for organic certificates (at **0.043**), and significant Levene’s test for nutrition apps and social marketing (Table A2). The Breusch-Pagan test was also significant for social marketing (***p* = 0.006**), but not for nutrition apps (*p* = 0.200). Regarding opinions about characteristics of addressees of healthy eating policies that matter for their effectiveness, all the assumptions were met with the exception of the Levene’s test for nutrition knowledge, BMI, and health status (Table A3). The Breusch-Pagan test was also significant for nutrition knowledge (***p* = 0.006**) and health status (***p* = 0.005**), but not for BMI (*p* = 0.705). As far as the assessment of credibility of selected types of information is concerned, the assumptions were met with the exception of interaction term of country and age for the list of ingredients, and Levene’s tests for health claims and organic certificates (Table A4). At the same time, the Breusch-Pagan test indicated no problem with heteroscedasticity for health claims (*p* = 0.513) and organic certificates (*p* = 0.099). To sum up, in the vast majority of cases, ANCOVAs assumptions were fulfilled.

Open-ended questions were analyzed with the identification of the most frequently occurring themes. Answering the open-ended questions was optional. A hybrid deductive–inductive codebook was developed. Deductive codes were derived from the NOURISHING framework and prior healthy-eating policy literature; inductive codes were added for themes emerging from the raw responses. The coding unit was the response; because respondents often mentioned several instruments, a single response could receive multiple codes. Regarding the open question about other instruments for healthy eating promotion, the answers were provided by 24 Polish respondents, 18 French respondents, 15 UK respondents, and 6 German respondents. As far as the open question about additional characteristics of audiences of healthy eating policies is concerned, the answers were provided by 7 Polish respondents, 4 French respondents, 8 UK respondents, and 6 German respondents. They were provided in the language of a given respondent (Polish, French, English, or German). Artificial intelligence (DeepSeek, version: DeepSeek-V4) was used to synthetize the qualitative findings. We verified the accuracy of the syntheses.

The share of women in our sample is very high—90.8%, with slight variation across countries: Poland: 87.5, France: 88.9, UK: 100.0, Germany: 92.3. The differences in the share of women were not significant (Chi^2^(3) = 3.571, *p* = 0.312).

The mean age of respondents was 42.6 years, and the median was slightly lower at 41 (Table 1). Age differed across countries with Polish respondents being the youngest and British—the oldest. Post-hoc tests with Bonferroni corrections showed significant differences: Poland vs. France (***p* = 0.006**), Poland vs. UK (***p* < 0.001**), Poland vs. Germany (***p* < 0.001**), France vs. UK (***p* < 0.001**).

The surveyed nutrition professionals live in various environments. The largest share (almost one third) live in large cities, whereas about one fifth live in rural areas (Table 2). The differences regarding place of residence were not significant across countries.

Over three quarters of the surveyed nutrition professionals reported running their own business and company (Table 3). Variation across countries was significant, as all UK respondents had their own business, and only a half of Polish nutrition professionals were self-employed. About a quarter of respondents had an employment contract. Again, international differences were large, with German subsample being composed to the largest extent by employees. It is worth noting that some nutrition professionals combined self-employment with being employed, especially in Western Europe, whereas a considerable share of Polish nutrition professionals had neither of these statuses, which may be related to their younger age and participation in education. 72.5% of all respondents reported having a website. These figures were the highest in the UK and France.

The structure of our sample was diverse in terms of the duration of professional experience as a dietitian (or nutritionist) (Table 4). The average experience in the profession amounted to 11.4 years overall, but it was more than twice as much in Germany. The shortest spans of professional experience were observed in Poland. Post-hoc tests with Bonferroni corrections showed that the following differences were significant: Poland vs. France (***p* = 0.004**), Poland vs. Germany (***p* < 0.001**), France vs. Germany (***p* = 0.001**), UK vs. Germany (***p* < 0.001**).

## 3. Results

The respondents were asked to evaluate the importance of selective instruments used to promote healthy eating (Table 5). A five-point scale with labels (meaningless, rather small, medium, rather big, very big) was applied and coded as 1–5. Overall, the surveyed nutrition professionals underlined the importance of nutrition education for children and adolescents, of the list of ingredients, and nutritional information placed on food labels, as all these measures were evaluated above 4. The remaining policy instruments were evaluated in the 3–4 range, which means medium to rather big importance. Relatively least important in this context were organic certificates, quality signs and cooking blogs.

In ANCOVAs adjusted for age and professional experience, the country effect was significant for social marketing campaigns (***p* = 0.014**), cooking blogs (***p* = 0.045**), nutrition apps (***p* = 0.008**), recommendations of scientific institutes (***p* = 0.015**), and information about the degree of product processing (***p* = 0.020**). However, after applying the Benjamini–Hochberg correction, no ANCOVAs meet the significance threshold.

In an open question, the respondents were also given the possibility to indicate other instruments for promoting healthy eating which they considered important. As far as Polish respondents are concerned, they emphasized the need for mandatory nutrition education for several specific groups. These include couples planning to have children, as well as young mothers, who play a crucial role in shaping the eating habits of future generations. They also stressed the importance of working directly with entire families to build long-term healthy habits. Furthermore, education should not be limited to children and adolescents; it should also extend to their parents, guardians, and teachers. Additional groups mentioned as needing targeted education were senior citizens and individuals living with obesity. Within schools, the proposals included introducing mandatory nutrition classes, ensuring that school canteens and vending machines offer healthy options, and engaging young people through project-based initiatives related to food in their local environment. Multiple suggestions focused on improving communication through media. The nutrition professionals recommended leveraging social media platforms like Instagram, TikTok, and YouTube to reach a wide audience, particularly young people and adults. They suggested using these channels to feature nutrition professionals who can provide real education and debunk myths, as well as influencers, including those documenting their own weight-loss journeys. To maximize impact, they proposed involving popular and respected personalities in public campaigns, thereby using the mechanism of social proof and trusted authorities. A crucial point raised was the need for dedicated fact-checking to counter the vast amount of health misinformation found online. The experts also expressed that professionals in the field should be given a larger platform to speak, arguing that it is a misunderstanding when nutrition articles in the media are written by journalists without specialized knowledge, which often leads to confusion. Other ideas included creating television programs dedicated to nutrition and launching public social campaigns focused on fighting health disinformation. On the systemic level, the nutrition professionals called for broader access to dietitians within primary healthcare clinics. They suggested that referrals and recommendations from general practitioners would help direct patients to the right specialists. An important underlying theme was the need to build public trust in qualified nutrition specialists. In terms of policy, the suggestions included implementing clearer country of origin labelling and highlighting locally sourced products to help consumers make informed choices. They also called for more precise standards regarding the composition and quality of food products. One respondent encapsulated this sentiment by stating that the best form of promotion is simply not misleading the consumer. Finally, the importance of direct, personal interaction was highlighted. The nutrition professionals stressed that having direct conversations with patients, explaining concepts, and answering their questions face-to-face remains a fundamental and effective tool for promoting healthy eating. To sum up, Polish respondents emphasized mandatory nutrition education for children and adolescents, but also for parents, teachers, pregnant couples, young mothers, seniors, and people with obesity. They frequently mentioned access to dietitians in primary care and trust in qualified professionals. Media-related suggestions included social media, TV programs, and fact-checking. Regulatory ideas were less frequent but included origin labelling and stricter composition standards.

In the subsample of French nutrition professionals, the following issues attracted the most attention: (1) education and skill-building, (2) accessibility to professionals and healthcare coverage, (3) communication, messaging, and media, and (4) regulation of marketing. The French nutrition professionals placed a strong emphasis on practical, experiential learning. They proposed therapeutic education focused on eating habits and food-related behavior, as well as education on taste to help people appreciate different flavors. A recurring theme was the importance of teaching children to cook and valuing culinary diversity. Several respondents mentioned organizing workshops for hands-on experimentation, such as cooking classes and gardening activities, which engage all five senses. These workshops should be designed for all age groups and adapted to various medical conditions. The nutrition professionals also suggested raising awareness about the ecological impact of food choices and informing people about the importance of eating local and seasonal produce while avoiding ultra-processed foods. A major concern was the unequal access to nutrition professionals. The nutrition professionals advocated for better accessibility to dietitians and nutritionists through reimbursement options beyond private health insurance, which they noted creates inequality. They proposed that consultations should be covered by the national social security system, employers, or organizations such as France Travail to ensure everyone can afford professional guidance. The experts highlighted the need for uniform and consistent nutritional messages to cut through the current fog of information that confuses the public. They recommended national multimedia campaigns involving television and medical professionals. Several nutrition professionals suggested using social media for awareness-raising, but insisted this should be done by qualified professionals rather than influencers. Information should start as early as primary school, with age-appropriate nutrition education. When it comes to product packaging, one respondent cautioned that too much information kills the information, suggesting instead to highlight basic food products and include simple recipe ideas. Regarding policy measures, the French nutrition professionals proposed several regulatory instruments. One striking suggestion was to place warning labels on unhealthy products, similar to the images and messages found on cigarette packets, with phrases such as “Too much sugar seriously harms your health” or “Too salty, too risky.” Another proposal was to heavily tax so-called toxic foods while clearly explaining why, place them in less visible sections of stores, and ban all advertising for these products. Specifically, the nutrition professionals called for improving children’s nutritional environment by prohibiting advertising aimed at children for all ultra-processed, fatty, salty, and sugary products. To sum up, French respondents stressed practical, experiential learning (cooking workshops, gardening, sensory education) and accessibility/reimbursement of dietitian services. They also called for national multimedia campaigns and stricter regulation of marketing, including warning labels similar to cigarette packets.

The UK respondents focused on the following themes: (1) education and skill-building, (2) healthcare system and professional involvement, (3) media, communication, and influencers, (4) regulation of labelling and marketing, and (5) community and retail environment. The UK nutrition professionals strongly emphasized the need for practical, lifelong education starting from an early age. They proposed compulsory cookery classes and nutrition lessons in schools, continuing all the way through from primary education. A specific suggestion was to provide nutrition education to mothers during antenatal visits, recognizing their crucial role in shaping early eating habits. For the general public, they called for factual, easy-to-implement educational resources and programs that avoid creating food stigma or snobbery, noting that current media streams often demonize entire food groups regardless of their nutritional value. Several respondents highlighted the role of healthcare professionals in promoting healthy eating. They suggested that GPs and other primary care practitioners should place greater emphasis on nutrition during consultations and actively promote referrals to qualified nutritionists and nutritional therapists. To improve accessibility, one dietitian proposed that nutritional therapy should be recognized and covered by health insurance companies, enabling more people to access these services and receive proper education. The NHS was called upon to lead real education campaigns in schools and local surgeries. The nutrition professionals expressed concern about the current media landscape, particularly the rise of social media influencers without formal nutrition training who launch supplements or promote specific ways of eating to large followings. Rather than dismissing this channel, they proposed a collaborative approach: dietitians should work alongside influencers and celebrity chefs to ensure that information shared on social media is science-based and evidence-based. They noted that both younger and older people increasingly turn to these platforms for nutritional advice. Other media instruments mentioned included billboard advertising, and newspaper campaigns. Many suggestions focused on regulatory and marketing measures. The nutrition professionals called for stricter regulation of health and nutrition claims made on food products, noting that the general public is often confused and misled by marketing language. They proposed placing warning labels on highly processed foods, similar to the warning signs found on cigarette packets, explicitly linking these products to chronic disease. Other ideas included improving product placement in supermarkets to give healthier foods more visibility, regulating supermarket advertising practices, and ensuring that information on food labelling is understood in the context of appropriate portion sizes. One respondent emphasized the need for policies that are achievable, simple, and cost-effective for the average person. Beyond schools and healthcare settings, the nutrition professionals mentioned the potential of community-based initiatives such as widespread availability of farmers’ markets to increase access to fresh, whole foods. They also highlighted the powerful influence of supermarkets in shaping consumer choices through advertising and product placement, calling for this influence to be redirected toward promoting healthier options. To sum up, UK respondents prioritized compulsory cookery and nutrition lessons from primary age, NHS-led education, and referrals to qualified nutritionists. They expressed strong concern about social media influencers without nutrition qualifications and proposed collaboration between dietitians and influencers. Regulatory suggestions included warning labels on highly processed foods and stricter control of health/nutrition claims.

Regarding the opinions of German nutrition professionals, the following suggestions were expressed: (1) education across the lifespan, (2) healthcare system and professional staffing, (3) regulatory measures in specific environments, and (4) health insurance and financial incentives. The German nutrition professionals placed strong emphasis on continuous, age-appropriate nutrition education for all population groups. They proposed teaching healthy eating from kindergarten all the way through to secondary school graduation, ensuring that children and young people receive consistent nutritional knowledge throughout their entire educational journey. This education should be supported by offering healthy options in school canteens. Beyond the school setting, they recommended cooking courses and group training sessions as practical instruments for building food skills. A specific focus was placed on providing nutritional counselling and support for pregnant women and their partners, recognizing the importance of early intervention. A key concern was the need for more qualified professionals in institutional settings. The nutrition professionals called for hiring more dietetic assistants in hospitals, clinics, and nursing homes to ensure that vulnerable populations receive appropriate nutritional care. This reflects a recognition that proper nutrition is essential not only for prevention but also for treatment and recovery in healthcare facilities. The respondents proposed targeted regulatory interventions to limit the availability of unhealthy products in certain settings. Specifically, they suggested restricting the availability of sweets and, most importantly, sugary drinks in places such as schools. This approach aims to create healthier food environments by reducing exposure to products high in added sugar, particularly for children and adolescents. An innovative instrument mentioned was the use of financial incentives through health insurance providers. The nutrition professionals proposed that health insurance companies should offer reduced premiums or contribution discounts to individuals who maintain a healthy lifestyle. This approach would reward positive behavior change and create a tangible financial motivation for adopting healthier eating habits. To sum up, German respondents emphasized lifespan nutrition education from kindergarten to secondary school, more dietetic assistants in hospitals/clinics/nursing homes, and regulatory restrictions in specific settings (e.g., sugary drinks in schools). They also mentioned health insurance incentives for healthy lifestyles.

Table 6 presents the final coding frequencies. Because responses could receive multiple codes, the total number of mentions exceeds the number of respondents. After reviewing all the responses from nutrition professionals across Poland, France, the UK, and Germany, the solution that was mentioned most frequently is nutrition education in schools and educational settings for children and young people. This theme appears consistently across all four countries, often with specific details about how and when this education should be delivered. The second most frequently mentioned solution was improving access to qualified nutrition professionals, whether through healthcare systems, insurance coverage, or institutional staffing. The third most frequent theme was regulating food marketing, labelling, and advertising, with specific proposals including warning labels on unhealthy products (compared to cigarette packets by both French and UK nutrition professionals), restricting advertising aimed at children, and clearer country of origin labelling. Examples of supporting quotations by theme and by country are shown in Table 7.

We asked the nutrition professionals to rate the level of use of selected healthy eating policies in their respective country (Table 8). Table 5 contains 14 instruments, while Table 8 and Table 9 refer to 12 instruments. We decided not to ask the respondents about the level of use of the list of ingredients and nutritional information on food labels, as their use is mandatory in the countries included in this study. The responses were measured in 5-point scales with labels (very big, rather big, average, rather small, little or none) and coded as 5-1.

Across all countries combined, the instruments perceived as most used were nutrition claims with a mean of 3.66, followed by nutrition apps for smartphones at 3.41, and quality signs on food labels at 3.28. Health claims scored 3.25 overall, while cooking blogs reached 3.24 and organic certificates 3.23.

The instruments perceived as least used across all countries were information about the degree of product processing with a mean of 2.44, followed by higher taxes on less healthy food products at 2.50. Nutrition education for children and adolescents scored 2.76 overall, while dietitian’s recommendations reached 2.94 and recommendations of scientific institutes scored 2.90.

In ANCOVAs adjusted for age and professional experience, the country effect was significant for quality signs on food labels (***p* = 0.007**) and nutrition apps (***p* = 0.024**). However, after Benjamini–Hochberg correction, no ANCOVAs remain significant.

Table 9 presents a comparison between how important European nutrition professionals find various healthy eating policy instruments and how much they feel these instruments are actually used in their country. The analysis uses *t*-tests for dependent samples, which means it compares the average scores for perceived “importance” and perceived “use” for each specific item to see if the difference between them is statistically significant.

The Benjamini–Hochberg-adjusted results match the raw conclusions: all the differences remain significant. Overall, the results reveal a significant gap between the importance placed on most policy instruments and their perceived use, with importance generally rated higher than use.

Starting with the instruments that show the most substantial differences, nutrition education for children and adolescents was rated as the single most important instrument, with an importance score of 4.59. However, it received one of the lowest perceived usage scores at 2.76. This massive gap is highly statistically significant, indicating that European nutrition professionals feel very strongly that education in schools is critical but is currently underutilized. Similarly, information about the degree of product processing was rated as very important, with a score of 3.92, but respondents felt this information is rarely available, giving it a usage score of only 2.44. This suggests a strong appetite for clearer systems that indicate how processed a food is.

Other instruments also show large gaps. For instance, dietitian’s recommendations are seen as very important, scoring 3.86, but the accessibility or visibility of these services is perceived as low, with a usage score of just 2.94. Likewise, higher taxes on less healthy food products received a moderate-to-high importance score of 3.46, yet respondents felt these are rarely implemented, reflected in the low usage score of 2.50. Health claims on products and recommendations of scientific institutes also followed this pattern, with importance significantly outweighing perceived use. Social marketing campaigns showed a significant but more moderate gap, with importance scored at 3.60 and use at 3.19.

For three instruments, the perceived use matches the importance placed on them. Nutrition claims, such as “low fat” or “high fiber”, have nearly identical perceived importance and perceived use scores of 3.67 and 3.66 respectively, suggesting the experts feel these claims are used about as much as they should be. Cooking blogs had both perceived importance and perceived use at 3.24, indicating that their availability is exactly in line with the expectations of nutrition professionals. Nutrition apps for smartphones also show no significant gap, with a perceived importance score of 3.45 and a perceived use score of 3.41.

Finally, two instruments show a slightly negative t-score, meaning perceived use is slightly higher than perceived importance, although these differences are not statistically significant. Organic certificates have a perceived use score of 3.23 compared to a perceived importance score of 3.17, while quality signs on food labels have a perceived use score of 3.28 versus a perceived importance score of 3.21.

Table 10 presents the opinions of nutrition professionals from four European countries (Poland, France, the UK, and Germany), regarding which characteristics of the target audience (addressees) matter most for the effectiveness of healthy eating policies. The nutrition professionals rated the importance of various demographic and personal characteristics, and a one-way ANCOVA, adjusted for age and professional experience, was conducted to determine if there were statistically significant differences in opinions between the four countries.

In ANCOVAs adjusted for respondent’s age and professional experience, the country effect was significant for age (of the policy addressee) (***p* = 0.041**), place of residence (urban vs. rural) (***p* = 0.041**), lifestyle (***p* = 0.030**), and nutrition knowledge (***p* = 0.008**). However, after applying Benjamini–Hochberg to all 11 variables in Table 8 at α = 0.05, there remain no significant differences in opinions about the characteristics.

The respondents were provided the opportunity to suggest additional characteristics of addressees of healthy eating policies that might influence their effectiveness. The nutrition professionals highlighted several individual psychological factors that influence how effectively people receive and act upon healthy eating messages. These include self-awareness and personality traits, which determine whether someone is receptive to nutritional advice. An individual’s mindset and their subjective perception of the importance of nutrition play crucial roles, as does their intrinsic motivation to change. The history of a person’s relationship with food is also important, including whether they have ever been on a diet before and their general attitude toward eating. Additionally, individual hobbies, interests, and personal preferences shape how people engage with nutritional guidance.

A person’s physiological state has a substantial impact on their receptivity to healthy eating messages. The nutrition professionals noted that pregnancy creates a window of increased interest in nutrition, which is why several respondents specifically mentioned the importance of targeting expectant mothers and their partners. Similarly, individuals who are already affected by diet-related diseases or who are overweight demonstrate greater interest in nutritional information compared to those who are currently healthy. Mental health status and dependencies such as alcohol addiction also affect a person’s ability to engage with and benefit from healthy eating promotion.

The family context emerged as a powerful influence on the effectiveness of nutrition policies. The nutrition professionals mentioned the family’s educational approach and the eating habits modelled within the home as key determinants. Familial health conditions and genetic predispositions can also motivate or discourage engagement with healthy eating. Beyond the family, an individual’s friendship circle and broader social group exert considerable influence, as people are shaped by the norms and behaviors of those around them. The presence or absence of children in a household also affects priorities and receptivity to certain messages.

Cultural background, ethnic origin, and religious practices all shape how people relate to food and nutrition. The nutrition professionals noted that individuals who follow specific dietary patterns due to religious requirements or personal choices such as veganism have distinct needs and motivations. Values and even political orientation were mentioned as factors that can influence whether someone trusts certain sources of nutritional information or accepts particular policy approaches.

Table 11 presents the opinions of nutrition professionals regarding the credibility of various types of nutrition and health-related information.

The nutrition professionals were asked to assess how trustworthy they find each information source, and a one-way ANCOVA, adjusted for age and professional experience, was conducted to determine if there were statistically significant differences in these credibility assessments between the four countries.

In ANCOVAs adjusted for age and professional experience, the country effect was significant for health claims (***p* = 0.002**), nutrition claims (***p* < 0.001**), social marketing campaigns (***p* < 0.001**), cooking blogs (***p* = 0.028**), and nutrition apps (***p* < 0.001**). The raw effects for health claims, nutrition claims, social marketing, nutrition apps, and cooking blogs remain significant after the Benjamini–Hochberg correction.

Post-hoc tests with Bonferroni corrections for multiple comparisons revealed statistically significant differences:

For health claims—between Poland and UK (***p* = 0.009**), Poland and Germany (***p* = 0.011**). The means adjusted for age and professional experience were as follows: Poland (3.658, 95% confidence interval (CI): 3.418–3.898), France (3.189, 95% CI: 2.915–3.464), UK (2.880, 95% CI: 2.507–3.252), Germany (2.728, 95% CI: 2.229–3.226).

For nutrition claims—between Poland and UK (***p* = 0.015**), between France and Germany (***p* < 0.004**), between UK and Germany (***p* < 0.001**). The means adjusted for age and professional experience were as follows: Poland (3.797, 95% CI: 3.541–4.054), France (3.295, 95% CI: 3.002–3.588), UK (3.006, 95% CI: 2.607–3.404), Germany (4.352, 95% CI: 3.819–4.886).

For social marketing—between Poland and France (***p* < 0.001**), between Poland and UK (***p* = 0.003**), between Poland and Germany (***p* = 0.003**). The means adjusted for age and professional experience were as follows: Poland (3.404, 95% CI: 3.149–3.659), France (2.501, 95% CI: 2.210–2.793), UK (2.489, 95% CI: 2.093–2.885), Germany (2.279, 95% CI: 1.748–2.809).

For cooking blogs—between Poland and France (***p* = 0.035**). The means adjusted for age and professional experience were as follows: Poland (2.983, 95% CI: 2.739–3.227), France (2.455, 95% CI: 2.177–2.734), UK (2.774, 95% CI: 2.395–3.152), Germany (2.343, 95% CI: 1.836–2.850).

For nutrition apps—between Poland and France (***p* < 0.001**), between Poland and UK (***p* < 0.001**), between Poland and Germany (***p* = 0.081**). The means adjusted for age and professional experience were as follows: Poland (3.579, 95% CI: 3.365–3.793), France (2.874, 95% CI: 2.629–3.118), UK (2.735, 95% CI: 2.403–3.066), Germany (2.925, 95% CI: 2.481–3.369).

In summary, nutrition professionals across Europe agree that the list of ingredients is the most credible source of information, followed closely by nutritional information. This consensus highlights the profession’s trust in factual, regulated, and non-marketing-based information. However, the results also reveal striking cross-country differences in professional skepticism. Polish nutrition professionals consistently rate the credibility of almost all information types higher than their European colleagues, suggesting they may have a more trusting disposition towards various information sources, including marketing campaigns and apps. In contrast, German and British nutrition professionals consistently show the most skepticism, particularly towards health claims, nutrition claims, and social marketing campaigns. This indicates that professional judgments about the trustworthiness of nutrition information are shaped by the national context in which nutrition professionals practice.

## 4. Discussion

### 4.1. Interpretation of Results

The findings of this international comparative study reveal insights into how nutrition professionals across four major European countries perceive healthy eating policies, their implementation, and the factors that influence their effectiveness.

One of the most striking findings of this study is the substantial gap between the importance that nutrition professionals attach to various healthy eating policy instruments and their perceived level of use across Europe. Nutrition education for children and adolescents emerged as the most highly valued instrument, with a mean importance score of 4.59, yet it received one of the lowest perceived usage scores at just 2.76. This discrepancy represents the largest gap among all instruments evaluated. This finding aligns with a growing body of evidence highlighting the critical role of early nutrition education. Nam et al. [16] demonstrated that school-based nutrition education programs can effectively enhance foundational nutrition knowledge among children, particularly in underserved populations. However, their research also cautioned that achieving sustained behavioral change requires longer interventions incorporating self-efficacy building strategies and environmental supports. Similarly, a systematic review of school-based health and nutrition interventions found that while such programs show promise in addressing malnutrition, their effectiveness depends on comprehensive, integrated approaches that extend beyond the classroom [17]. The consistent emphasis on nutrition education across all four countries in our qualitative data, with nutrition professionals proposing compulsory cookery classes, nutrition lessons throughout the educational journey, and targeted education for specific groups including pregnant women and seniors, reflects what Nguyen et al. [4] identified as essential enablers of successful policy implementation: stakeholder engagement and a whole-school approach. Their systematic review of food environment policies implemented at scale found that stakeholder engagement and prioritization of policies were among the most commonly cited enablers of successful implementation.

Information about the degree of product processing showed the second largest implementation gap, with importance rated at 3.92 but perceived use at only 2.44. This finding suggests that European nutrition professionals see considerable room for improvement in how food processing information is communicated to consumers. The qualitative data from French and UK nutrition professionals particularly emphasized this point, with several respondents proposing warning labels on unhealthy products comparable to those found on cigarette packets. This desire for greater transparency may reflect growing professional concern about ultra-processed foods and their health impacts.

A recurring theme throughout our findings is the presence of cross-national differences in how nutrition professionals evaluate both the importance of various policies and the credibility of different information sources. These differences were particularly pronounced for nutrition knowledge as a characteristic of policy addressees, with Polish nutrition professionals rating this factor most highly and German nutrition professionals assigning it the lowest importance. Dierckens et al. [18] provide a useful framework for understanding such cross-national variations. Their multilevel analysis of adolescent dietary behaviors across 21 European countries demonstrated that country-level sociocultural context is associated with socioeconomic inequalities in dietary behaviors. The credibility assessments in our study revealed particularly striking patterns. German nutrition professionals placed the highest trust in nutrition claims (4.38) but were among the most skeptical of health claims (2.54). British nutrition professionals consistently showed the highest levels of skepticism across multiple information types, while Polish nutrition professionals consistently rated credibility higher than their European colleagues. These patterns may reflect different national regulatory traditions, historical experiences with food marketing, or cultural orientations toward institutional information sources. Hinojosa-Nogueira et al. [19] recently demonstrated significant differences in dietary behaviors and portion size selection between Spanish and German adults, highlighting how cultural preferences and dietary guidelines shape national eating patterns and, by extension, may influence professional perspectives on nutrition information. Polish experts value clear information systems like Nutri-Score, but widespread supplement use and health literacy issues may affect opinions on selective tools [20,21]. France promotes healthy eating through public campaigns and Nutri-Score, but cultural values (pleasure vs. health) influence dietitians’ priorities [22,23,24]. UK dietitians’ decisions are shaped by pragmatism and individual responsibility, leading to diverse opinions on intervention strategies [24,25]. German dietitians are legally recognized and focus on counseling, with regulatory tools like nutrient profiling discussed for health claims [26,27]. In summary, differences in opinions of nutrition professionals across these countries are shaped by cultural attitudes, professional roles, health literacy, and the adoption of policy tools like Nutri-Score. These factors interact to create distinct perspectives on the importance of selective instruments for promoting healthy eating.

The nutrition professionals in our study identified lifestyle, income, nutrition knowledge, and education level as the most important characteristics of target audiences that influence the effectiveness of healthy eating policies. This finding resonates with broader public health literature. Houghtaling et al. [28] found that engaging dedicated community partners and community members, and starting small to ensure success, were highly ranked implementation strategies for healthy eating and active living policy changes. Their research emphasizes the importance of tailoring interventions to specific community contexts and populations. Consumers’ lack of nutritional knowledge about food can prevent the Nutri-Score label from being used appropriately [29]. Nutritionally knowledgeable and health-conscious consumers discount health labels on healthy products [30].

The family context emerged as a particularly powerful influence, with nutrition professionals mentioning family educational approaches, eating habits modelled within the home, and familial health conditions as key determinants. This finding aligns with Palmer et al. [31], whose systematic review of policy, systems, and environmental changes in child nutrition programs found that interventions incorporating family and community components were more likely to achieve sustained improvements in dietary behaviors.

Higher taxes on less healthy food products showed a substantial implementation gap (perceived importance 3.46, perceived use 2.50), indicating that nutrition professionals across Europe see untapped potential in fiscal measures. This finding is consistent with the NOURISHING framework developed by the World Cancer Research Fund International, which identifies fiscal policies as a key component of comprehensive approaches to promoting healthy diets [3]. The qualitative data from French nutrition professionals specifically proposed heavy taxation of so-called toxic foods while clearly explaining why, placing them in less visible sections of stores, and banning all advertising for these products. UK nutrition professionals similarly called for stricter regulation of health and nutrition claims and proposed warning labels on highly processed foods. These professional perspectives align with the barriers and enablers identified by Nguyen et al. [4], who found that concern over profitability and revenue was among the most commonly cited barriers to policy implementation, while policy-level support in the form of information, guidance, and training was a key enabler.

The UK nutrition professionals expressed particular concern about social media influencers without formal nutrition training who launch supplements or promote specific ways of eating to large followings. Rather than dismissing this channel entirely, they proposed a collaborative approach whereby nutrition professionals work alongside influencers and celebrity chefs to ensure that information shared on social media is science-based. This pragmatic response acknowledges the reality identified by Grunert et al. [32] that people use diverse sources of nutrition information, and that the credibility of these sources varies considerably. The credibility assessments in our study provide empirical grounding for these concerns. Cooking blogs received the lowest credibility ratings among all information types (total mean 2.73), with particularly low scores in France (2.47), and Germany (2.38). Social marketing also received low credibility ratings overall. These findings suggest that nutrition professionals view many contemporary information sources with considerable skepticism, while maintaining strong trust in traditional, regulated sources such as ingredient lists and nutritional information panels.

### 4.2. Study Limitations

Several methodological considerations should be borne in mind when interpreting these findings. First, the sample sizes varied considerably across countries, with 56 respondents in Poland, 36 in France, 26 in the UK, and only 13 in Germany. The small German subsample, in particular, limits the generalizability of findings from that country and may have affected the statistical power to detect differences involving Germany.

Second, significant differences in respondent characteristics across countries, particularly age and years of professional experience, may have influenced the findings. Polish respondents were significantly younger and had fewer years of professional experience than their counterparts in other countries. Younger professionals may have different educational backgrounds, professional socialization experiences, and exposure to contemporary nutrition science that shape their perspectives. Although we addressed our survey to dietitians, we also allowed nutritionists to participate. We did not measure the share of nutritionists in our national samples, which may be a source of bias.

Third, the study relied on self-reported perceptions of policy use rather than objective measures of policy implementation. While nutrition professionals are well-positioned to observe the policy environment in their countries, their perceptions may not perfectly align with actual policy implementation levels.

Fourth, the cross-sectional design captures perspectives at a single point in time and cannot track how opinions may evolve in response to policy changes or shifting professional contexts. Longitudinal research would be valuable for understanding how dietitians’ views respond to new policy initiatives and changing information environments.

### 4.3. Policy Implications

These findings have several implications for the development of healthy eating policies. The consistent implementation gap identified across multiple policy instruments suggests a need for research that examines not only whether policies are in place but also how effectively they are implemented and communicated. Understanding how to select and operationalize implementation strategies in alignment with implementation science recommendations remains an important research priority.

The cross-national differences observed in this study underscore the importance of context-sensitive policy development. Policies that are effective in one national context may not translate directly to another, given differences in cultural orientations toward food, institutional structures for healthcare and education, and historical experiences with nutrition policy.

The emphasis of our respondents on nutrition education, particularly for children and adolescents, suggests that this should remain a priority for policy development and funding. Investments in school-based nutrition education should be sustained and comprehensive rather than episodic.

Finally, the concerns expressed by nutrition professionals about their professional identity and the challenge of misinformation point to the need for strategies that strengthen the role of qualified nutrition professionals in public discourse. This might include greater involvement of dietitians in media communications, partnerships with influencers to ensure accurate information reaches diverse audiences, and public education about the qualifications and expertise of registered dietitians compared to non-qualified sources of nutrition advice.

## 5. Conclusions

This study provides comprehensive evidence of an implementation gap in European healthy eating policies as perceived by nutrition professionals. The profession strongly endorses educational interventions for children and adolescents, transparency tools such as processing information, and professional dietary advice, yet perceives these instruments as substantially underutilized.

Cross-national differences in professional perspectives are pronounced, with Polish nutrition professionals consistently rating the credibility of information sources more highly than their European colleagues, while British and German nutrition professionals often display greater skepticism. These differences likely reflect the interplay of national policy histories, cultural orientations toward food and health, and professional socialization patterns. At the same time, there were no significant country effects for the evaluation of policy importance and use as well as for characteristics of target consumers that might affect the effectiveness of healthy eating policies.

The findings underscore the need for context-sensitive policy development that attends not only to the design of healthy eating policies but also to their implementation, the characteristics of target audiences, and the information environments in which people make food choices. Engaging nutrition professionals as key stakeholders in policy development and implementation could help bridge the gap between policy intentions and realized impacts, drawing on their expertise in understanding what works for whom and under what conditions.

## Figures and Tables

**Table 1:** The age of respondents.

Age	Total	Poland	France	UK	Germany	F	*p*
Mean	42.63	35.86	42.75	52.96	50.85	**22.303**	**<0.001**
Standard Deviation	11.78	8.51	10.80	10.39	9.33		
Minimum	23	23	24	31	36		
First Quartile	34	30	36	44	43		
Median	41	35.5	41.5	51.5	48		
Third Quartile	50	41	49.5	62	60.5		
Maximum	75	72	72	75	65		

Note: Bold is used to highlight statistically significant differences at *p* < 0.05.

**Table 2:** Place of residence—size of town (%).

Place of Residence	Total	Poland	France	UK	Germany	Chi^2^(9)	*p*
Village	21.4	19.6	27.8	15.4	23.1	13.210	0.153
Town < 50,000 inhabitants	24.4	17.9	38.9	19.2	23.1		
City 50–500,000 inhabitants	22.1	21.4	13.9	26.9	38.5		
City > 500,000 inhabitants	32.1	41.1	19.4	38.5	15.4		

**Table 3:** Characteristics of respondents (%).

Characteristics	Total	Poland	France	UK	Germany	Chi^2^(3)	*p*
Running one’s own business or company	75.6	53.6	97.2	100.0	61.5	**33.615**	**<0.001**
Employment contract	25.2	17.9	19.4	26.9	69.2	**15.650**	**<0.001**
Website	72.5	58.9	86.1	88.5	61.5	**12.630**	**0.006**

Note: Bold is used to highlight statistically significant differences at *p* < 0.05.

**Table 4:** Years of professional experience as a dietitian (or nutritionist).

Professional Experience	Total	Poland	France	UK	Germany	F	*p*
Mean	11.42	7.21	13.50	11.35	23.92	**14.926**	**<0.001**
Standard Deviation	9.68	5.45	10.86	8.39	11.17		
Minimum	0	0	2	1	5		
First Quartile	4	3	5.25	4	14.5		
Median	9	5.5	12	10	25		
Third Quartile	16	10	18	16.5	32		
Maximum	50	25	50	30	42		

Note: Bold is used to highlight statistically significant differences at *p* < 0.05.

**Table 5:** Opinions about the importance of selective instruments for the effective promotion of healthy eating (one-way ANCOVAs), adjusted for age and professional experience.

Instruments	Total	Poland	France	UK	Germany	F	*p*	BH-Adjusted *p*
Health claims	3.69	3.84	3.47	3.77	3.46	0.645	0.588	0.592
Nutrition claims	3.67	3.68	3.42	4.00	3.69	1.757	0.159	0.288
List of ingredients	4.19	4.29	4.03	4.27	4.08	0.637	0.592	0.592
Nutritional information	4.11	4.32	3.89	4.08	3.92	1.634	0.185	0.288
Social marketing campaigns	3.60	3.73	3.11	3.81	3.92	**3.681**	**0.014**	0.070
Cooking blogs	3.24	3.46	3.00	3.04	3.31	**2.754**	**0.045**	0.126
Nutrition apps for smartphones	3.45	3.82	3.17	3.00	3.54	**4.134**	**0.008**	0.070
Organic certificates	3.17	2.98	3.47	3.19	3.08	2.261	0.085	0.198
Quality signs on food labels	3.21	3.21	3.56	2.88	2.92	1.660	0.179	0.288
Recommendations of scientific institutes	3.38	3.54	3.72	2.58	3.38	**3.648**	**0.015**	0.070
Dietitian’s recommendations	3.86	3.89	4.08	3.38	4.08	0.840	0.475	0.592
Higher taxes on less healthy food products	3.46	3.25	3.47	3.62	4.00	0.640	0.590	0.592
Nutrition education for children and adolescents	4.59	4.64	4.44	4.77	4.38	0.746	0.527	0.592
Information about the degree of product processing	3.92	3.77	4.28	4.08	3.31	**3.378**	**0.020**	0.070

Notes: unadjusted means are displayed; bold is used to highlight statistically significant differences at *p* < 0.05.

**Table 6:** Frequency of themes in open-ended responses, by country.

Code	Theme	PL (*n* = 24)	FR (*n* = 18)	UK (*n* = 15)	DE (*n* = 6)	Total
T1	School/child/adolescent nutrition education and food skills	14	9	9	4	36
T2	Access to qualified nutrition professionals and services	7	7	6	3	23
T3	Regulation of food marketing, labelling, advertising, and claims	3	6	7	2	18
T4	Family and life-course education	5	3	3	3	14
T5	Media, digital communication, and misinformation	7	4	5	0	16
T6	Community and retail food environment	2	4	4	3	13
T7	Fiscal/insurance incentives	0	2	1	2	5
T8	Direct counselling/personal interaction	2	0	0	0	2
T9	Other/none/don’t know	3	1	1	0	5

Note: Multiple codes per response; totals exceed country *n*.

**Table 7:** Examples of quotations from open-ended responses, by theme and by country.

Country	Theme	Quotation
Poland	T1	“Obowiązkowe zajęcia w szkołach z edukacji żywieniowej.” [“Mandatory nutrition education classes in schools.”]
	T2	“Szerszy dostęp do dietetyków w przychodniach POZ.” [“Greater access to dietitians in primary care clinics.”]
	T4	“obowiązkowa edukacja żywieniowa małżeństw planujących potomstwo, praca z rodziną nad nawykami żywieniowymi.” [“Mandatory nutrition education for couples planning to have children; working with families on dietary habits.”]
	T5	“Media społecznościowe—Instagram.” [“Social media—Instagram.”]
	T6	“Zdrowe opcje w stołówkach, sklepikach szkolnych, bufetach pracowniczych…” [“Healthy options in canteens, school shops, employee cafeterias…”]
	T8	“Przede wszystkim bezpośrednie rozmowy z pacjentami—wyjaśnianie i odpowiedzi na zadawane pytania.” [“Above all, direct conversations with patients—providing explanations and answering questions.”]
France	T1	“éducation nutritionnelle a l’école.” [“Nutritional education in schools.”]
	T2	“une meilleure accessibilité aux diététiciens nutritionniste avec une remboursement possible…” [“Better access to nutritionists/dietitians, with potential reimbursement…”]
	T3	“Trop de sucre nuit gravement à votre santé OU Trop salé, trop risqué…” [“Too much sugar seriously harms your health OR Too much salt, too much risk…”]
	T5	“Uniformité des messages car brouillard d’informations pour le grand public.” [“Consistent messaging, as there is an information overload for the general public.”]
UK	T1	“Cooking lessons and nutrition classes at schools all the way through from primary age.”
	T2	“Nutritional therapy should be recognised by health insurance companies so that more people can access these services…”
	T3	“the link to chronic disease on junk processed food packages (similar to warning signs on cigarette packets).”
	T5	“Social media influencers (often without any nutrition training/qualifications) with big followings…”
	T6	“Supermarkets and the way they market/advertise foods.”
Germany	T1/T6	“Gesunde Ernährung vom Kindergarten bis zum Abitur in Schulen unterrichten und in Kantinen anbieten.” [“Teach healthy nutrition in schools—from kindergarten through to the final year of secondary school—and offer it in canteens.”]
	T2	“Mehr Diätassistenten in Krankenhäusern, Kliniken und Altenheimen einstellen.” [“Hire more dietitians in hospitals, clinics, and nursing homes.”]
	T3	“Eingrenzen der Verfügbarkeit von Süßigkeiten und vor allem von gezuckerten Getränken, z.B. in Schule.” [“Restrict the availability of sweets and, above all, sugary drinks—for example, in schools.”]
	T7	“Angebote der Krankenkassen zu Beitragsermäßigungen bei gesunder Lebensweise.” [“Health insurance provider offers for premium reductions based on a healthy lifestyle.”]

**Table 8:** Opinions about the level of use of selected healthy eating policy instruments in one’s country (one-way ANCOVAs), adjusted for age and professional experience.

Instruments	Total	Poland	France	UK	Germany	F	*p*	BH-Adjusted *p*
Health claims	3.25	3.05	3.55	3.46	3.15	1.823	0.146	0.360
Nutrition claims	3.66	3.55	3.72	3.62	4.00	1.380	0.252	0.360
Social marketing campaigns	3.19	3.04	3.47	3.23	3.00	1.323	0.270	0.360
Cooking blogs	3.24	3.34	3.22	3.23	2.85	0.441	0.724	0.724
Nutrition apps for smartphones	3.41	3.52	3.69	3.12	2.77	**3.253**	**0.024**	0.144
Organic certificates	3.23	3.27	3.42	2.96	3.08	1.441	0.234	0.360
Quality signs on food labels	3.28	3.30	3.72	2.77	3.00	**4.211**	**0.007**	0.084
Recommendations of scientific institutes	2.90	3.02	3.03	2.38	3.08	1.742	0.162	0.360
Dietitian’s recommendations	2.94	2.98	3.06	2.69	2.92	0.446	0.720	0.724
Higher taxes on less healthy food products	2.50	2.61	2.33	2.69	2.15	2.294	0.081	0.324
Nutrition education for children and adolescents	2.76	2.71	3.00	2.54	2.69	0.608	0.611	0.724
Information about the degree of product processing	2.44	2.46	2.78	2.04	2.23	1.642	0.183	0.360

Notes: unadjusted means are displayed; bold is used to highlight statistically significant differences at *p* < 0.05.

**Table 9:** Comparison of perceived importance attached to healthy eating policy instruments with assessment of their use in one’s country (*t*-tests for dependent samples across Europe).

Instruments	Perceived Importance	Perceived Use	t	*p*	BH-Adjusted *p*
Health claims	3.69	3.25	**3.792**	**<0.001**	**<0.001**
Nutrition claims	3.67	3.66	0.140	0.889	0.970
Social marketing campaigns	3.60	3.19	**2.944**	**0.004**	**0.007**
Cooking blogs	3.24	3.24	0.000	1.000	1.000
Nutrition apps for smartphones	3.45	3.41	0.359	0.720	0.864
Organic certificates	3.17	3.23	−0.631	0.529	0.706
Quality signs on food labels	3.21	3.28	−0.695	0.488	0.706
Recommendations of scientific institutes	3.38	2.90	**4.593**	**<0.001**	**<0.001**
Dietitian’s recommendations	3.86	2.94	**8.716**	**<0.001**	**<0.001**
Higher taxes on less healthy food products	3.46	2.50	**7.393**	**<0.001**	**<0.001**
Nutrition education for children and adolescents	4.59	2.76	**13.984**	**<0.001**	**<0.001**
Information about the degree of product processing	3.92	2.44	**11.924**	**<0.001**	**<0.001**

Notes: unadjusted means are displayed; bold is used to highlight statistically significant differences at *p* < 0.05.

**Table 10:** Opinions about characteristics of addressees of healthy eating policies that matter for their effectiveness (one-way ANCOVAs), adjusted for age and professional experience.

Characteristics	Total	Poland	France	UK	Germany	F	*p*	BH-Adjusted *p*
Sex	3.63	3.88	3.42	3.38	3.62	1.961	0.123	0.193
Age	3.98	4.30	3.72	3.96	3.38	**2.835**	**0.041**	0.113
Number of people in the household	3.23	3.21	3.33	3.12	3.23	0.388	0.762	0.838
Place of residence—city/village	3.50	3.86	3.25	3.42	2.85	**2.835**	**0.041**	0.113
Education level	4.16	4.11	4.19	4.23	4.15	0.072	0.975	0.975
Income	4.28	4.36	4.19	4.35	4.08	0.522	0.668	0.816
Occupation performed	3.65	3.55	3.89	3.54	3.62	0.885	0.451	0.620
Lifestyle	4.40	4.63	4.22	4.27	4.15	**3.085**	**0.030**	0.113
Nutrition knowledge	4.37	4.68	4.11	4.35	3.77	**4.151**	**0.008**	0.088
Body Mass Index (BMI)	3.31	3.52	3.03	3.31	3.15	2.310	0.080	0.152
Health condition	3.95	4.18	3.78	3.85	3.62	2.280	0.083	0.152

Notes: unadjusted means are displayed; bold is used to highlight statistically significant differences at *p* < 0.05.

**Table 11:** Assessment of credibility of selected types of information (one-way ANCOVAs), adjusted for age and professional experience.

Types of Information	Total	Poland	France	UK	Germany	F	*p*	BH-Adjusted *p*
Health claims	3.28	3.75	3.17	2.81	2.54	**5.428**	**0.002**	**0.003**
Nutrition claims	3.56	3.80	3.31	2.96	4.38	**7.880**	**<0.001**	**<0.001**
List of ingredients	4.21	4.27	4.19	4.00	4.38	0.364	0.779	0.779
Nutritional information	4.11	4.30	4.06	3.73	4.15	1.955	0.124	0.186
Social marketing campaigns	2.86	3.38	2.50	2.54	2.31	**9.235**	**<0.001**	**<0.001**
Cooking blogs	2.73	3.00	2.47	2.69	2.38	**3.146**	**0.028**	**0.050**
Nutrition apps for smartphones	3.15	3.52	2.92	2.69	3.15	**8.238**	**<0.001**	**<0.001**
Organic certificates	3.53	3.36	3.78	3.65	3.31	1.780	0.154	0.199
Quality signs on food labels	3.11	3.21	3.22	2.88	2.85	1.192	0.316	0.355

Notes: unadjusted means are displayed; bold is used to highlight statistically significant differences at *p* < 0.05.

## Data Availability

The original data presented in the study are openly available in Zenodo at https://doi.org/10.5281/zenodo.18141494.

## Appendix A. Verification of Assumptions for ANCOVAs

**Table A1:** Verification of assumptions for one-way ANCOVAs, adjusted for age and professional experience, concerning opinions about the importance of selective instruments for the effective promotion of healthy eating.

Instruments	Country-Age Interaction *p*	Country- Experience Interaction *p*	Levene’s *p*
Health claims	0.548	0.836	0.557
Nutrition claims	0.931	0.997	0.475
List of ingredients	0.890	0.941	0.837
Nutritional information	0.934	0.960	0.122
Social marketing campaigns	0.124	0.120	0.973
Cooking blogs	0.832	0.544	0.176
Nutrition apps for smartphones	0.742	0.731	0.876
Organic certificates	0.330	0.117	0.128
Quality signs on food labels	0.879	0.744	0.127
Recommendations of scientific institutes	0.571	0.753	0.573
Dietitian’s recommendations	0.213	0.378	0.216
Higher taxes on less healthy food products	0.595	0.788	0.396
Nutrition education for children and adolescents	0.334	0.742	**0.002**
Information about the degree of product processing	0.930	0.915	0.429

Note: Bold is used to highlight a statistically significant test result at *p* < 0.05.

**Table A2:** Verification of assumptions for one-way ANCOVAs, adjusted for age and professional experience, concerning opinions about the level of use of selected healthy eating policy instruments in one’s country.

Instruments	Country-Age Interaction *p*	Country- Experience Interaction *p*	Levene’s *p*
Health claims	0.966	0.962	0.169
Nutrition claims	0.443	0.993	0.203
Social marketing campaigns	0.867	0.513	**0.009**
Cooking blogs	0.278	0.682	0.792
Nutrition apps for smartphones	0.687	0.649	**<0.001**
Organic certificates	0.499	**0.043**	0.226
Quality signs on food labels	0.335	0.058	0.896
Recommendations of scientific institutes	0.453	0.657	0.678
Dietitian’s recommendations	0.087	0.821	0.119
Higher taxes on less healthy food products	0.664	0.436	0.394
Nutrition education for children and adolescents	0.173	0.466	0.887
Information about the degree of product processing	0.099	0.735	0.751

Note: Bold is used to highlight statistically significant test results at *p* < 0.05.

**Table A3:** Verification of assumptions for one-way ANCOVAs, adjusted for age and professional experience, concerning opinions about characteristics of addressees of healthy eating policies that matter for their effectiveness.

Characteristics	Country-Age Interaction *p*	Country- Experience Interaction *p*	Levene’s *p*
Sex	0.176	0.230	0.540
Age	0.941	0.105	0.581
Number of people in the household	0.224	0.622	0.095
Place of residence—city/village	0.115	0.497	0.416
Education level	0.684	0.885	0.456
Income	0.147	0.682	0.059
Occupation performed	0.465	0.388	0.528
Lifestyle	0.744	0.596	0.348
Nutrition knowledge	0.848	0.788	**0.040**
Body Mass Index (BMI)	0.730	0.638	**0.029**
Health condition	0.772	0.317	**<0.001**

Note: Bold is used to highlight statistically significant test results at *p* < 0.05.

**Table A4:** Verification of assumptions for one-way ANCOVAs, adjusted for age and professional experience, concerning assessment of credibility of selected types of information.

Types of Information	Country-Age Interaction *p*	Country- Experience Interaction *p*	Levene’s *p*
Health claims	0.953	0.852	**0.001**
Nutrition claims	0.488	0.689	0.058
List of ingredients	**0.037**	0.215	0.279
Nutritional information	0.215	0.142	0.137
Social marketing campaigns	0.976	0.911	0.380
Cooking blogs	0.752	0.761	0.466
Nutrition apps for smartphones	0.142	0.087	0.197
Organic certificates	0.624	0.800	**0.040**
Quality signs on food labels	0.202	0.734	0.374

Note: Bold is used to highlight statistically significant test results at *p* < 0.05.
